# Lining Root-Canals

**Published:** 1896-09

**Authors:** L. P. Bethel

**Affiliations:** Kent, Ohio


### THE DENTAL REGISTER.

VOL. L.] SEPTEMBER, 1896. [No. 9.

### COMMUNICATIONS.

### Lining Root-Canals.

BY L. P. BETHEL, D.D.S , M.D., KENT, OHIO.

Abstract of a paper read before the American Dental Association, at Saratoga, New York, August, 1896.

In the treatment of teeth with devitalized pulps, a medicament that not only sterilizes the contents of the root-canal, but leaves behind an antiseptic deposit which prevents the subsequent development of micro-organisms, would be an ideal disinfectant.

With this thought in mind I began a series of experiments, some months ago, taking nitrate of silver for the first agent.

We know how useful this salt has been in the treatment of certain superficial cavities in the teeth of adults and various cavities in the teeth of children, preventing decay as long as the discoloration remains. If in this location, where it is exposed to the varying conditions of the oral fluids, it will prevent subsequent decay for a considerable time, why should it not remain unchanged for a much longer period when sealed within a root-canal and. remain, perhaps, as a permanent barrier to the development of germs?

Repeated attempts at pumping it into the canal by means of wooden points, broaches, etc., proved unsatisfactory, for the silver nitrate solution would not go beyond the point of penetration of the broach, and the cases most desired to treat were small, branching or tortuous canals, where it was impossible to pass even a broach. By the aid of cataphoresis, however, the silver nitrate was forced beyond where the broach extended, into small canals, etc., as these specimens show. Microscopic examination shows that the nitrate of silver is forced, by means of cataphoresis, to a greater depth into the tubuli of the dentine, more thoroughly sealing them than when applied to the surface by ordinary mechanical means.

In the preliminary experiments out of the mouth, the silver nitrate was used in connection with various agents, such as sulphate of soda, 1 percent $H_2SO_4$, etc., but the silver being itself a good conductor of electricity, it was found most satisfactory when used alone in an aqueous solution made from distilled water, to avoid all organic material. Various strengths were employed from 10 percent to a saturated solution, those giving the best results being from 40-percent to a 75-percent solution.

The process of application is a simple one; adjust the rubber dam, and if the crown of the tooth needs protection from discoloration, apply a thin coating of melted wax to the interior surface. Next apply the silver-nitrate solution to the canal by means of a wooden tooth-pick or any other suitably-shaped piece of wood, pump it downward into the canal as thoroughly as possible, place the electrode into the pulp-canal opening, then a pellet of cotton saturated with the nitrate solution around the electrode at the orifice of the canal, and the electricity does the rest.

The electric current turns the cotton first a light green color, which grows darker until almost black, and serves as an indicator. The time of application will vary according to the condition of the root-canal—whether well opened, its size, strength of current, and percent solution of the silver nitrate. The higher percent the solution the better conductor it makes, and the quicker it is deposited. From one to five minutes seems to be ample time.

After removing the electrode, cleanse the pulp-cavity and canals as well as possible, with diluted ammonia to neutralize the nitric acid set free, and also to hasten the darkening of the nitrate of silver.

In the majority of practical cases I have been using the nitrate after the root-canal has been sterilized, although in several cases it was used without previous sterilization, the cavity sealed and no after-trouble experienced.

This root-canal lining is not advocated for all teeth; indeed, the practitioner must use judgment in its application. It would not be advisable in the anterior teeth on account of discoloration, or teeth where the foramen is large, as teeth not fully developed and others, on account of forcing it through the apex of its root. Just what would result from such an accident I am unable to state from practical experience. I have tried to force the solution through the apex of a normal root, out of the mouth, but in every instance it has penetrated just through the foramen and stopped, due, possibly, to forming an albuminate when coming in contact with tissues at the end of the root, and thus limiting its own action.

The object of these experiments is to find a means of treating root-canals that are too small to admit a broach, those branching or tortuous, those in flat-rooted teeth, etc., where it is doubtful about inserting a protecting root-filling, If such root-canals are thoroughly lined with the nitrate solution, and it penetrates somewhat into the tubuli, as it does, the probability is that there will be no subsequent trouble, even though the root-filling should be defective. And, indeed, it is a question if root-filling would be necessary at all, especially in small canals.

Roots treated by this process out of the mouth, when filed, reveal the outlines of the canals, their restrictions, obstructions, and unlooked for branches that probably would not be found in ordinary root-treatment and filling, and left, perhaps, as a harbor for bacteria, in which to multiply and cause subsequent trouble.

This is only the beginning of a series of experiments in this direction. What the future may disclose time alone will tell.

DISCUSSION.

Dr. Abbott, New York, said that cataphoresis takes too much time. He uses chloride of zince, and fills with oxychloride. He thought the silver nitrate would permeate the tubules and become a source of danger to the cementum and pericementum.

Dr. L. L. Barber, Toledo, O., said that this treatment had proved satisfactory to him where other means had failed. He cited a case of a lower third molar, abscessed, that had resisted repeated attempts at treatment with various disinfectants, the tooth becoming painful after the dressings had been sealed in. One application of silver nitrate solution cataphoretically, as advocated in the paper, was used, the cavity sealed, and no inconvenience to the patient has been experienced since the operation.

Dr. Ambler, Cleveland, said that while he was experimenting with nitrate under amalgam fillings, Dr. Bethel suggested its use for root-canals, and together they made some preliminary experiments. He had since operated on cases in the mouth and no trouble has been experienced. He does not operate on roots having a large foramen, but where the canals are small and it is almost impossible to pass a broach. Cataphoresis drives the medicament deeper into the tubules than when locally applied, and this is an advantage. In the root-canal operated on with nitrate of silver you have an insoluble compound sealing the tubules and which can not be penetrated by anything from outside. It is not intended for teeth of children or where the foramen is large. It is not claimed that the use of silver nitrate is new, but this particular application of it certainly is. He has used it also with good results under amalgam fillings. There can be no subsequent decay as long as the dark deposit remains. He does not ask any one to use this method if they do not desire to do so, but he has had good results from its use.

Dr. Stephan, Cleveland, does not think that nitrate of silver should be used under any filling where there is a live pulp, on account of the liability of its causing the pulp to die.

Dr. B. Holly Smith, Baltimore, said he was very much pleased with the paper. This was the beginning of a series of experiments in the right direction, and they should be encouraged. He thought the idea of cataphoresis taking too much time, as expressed by Dr. Abbott, was entirely out of place. Let the operator have two chairs, if need be, and a competent assistant to operate the cataphoric machine. He asked how it was that the current of electricity would carry the nitrate along a tortuous canal?

Dr. Jos. Head, Philadelphia, said that as the canal was much larger than the tubules, it contained a great amount of moisture, and was, therefore, a better conductor of the electricity which would flow in the line of least resistance.

Dr. James Truman, Philadelphia, said that nitrate of silver being a strong antiseptic would prevent the development of germs, but it would discolor the tooth-substance. It would be carried into the tubules by osmosis and where would its limitations be, in the pulp-cavity or in the cementum? He had applied nitrate of silver to tooth-substance and found that it penetrated into the tubules. He preferred to use a medicament that would not discolor, and recommended chloride of zinc. Its application should not be by cataphoresis, however, for that would drive it through the tubules and would be apt to prove dangerous to the cementum or pericementum.

Dr. M. L. Rhein, New York, thinks that if an escharotic should be used that zinc chlorid offers superior advantages. We should use cataphoresis carefully, for the electric current reduces the medicaments to their nascent state.

Dr. J. Taft, Cincinnati, thought that the gentleman had an exaggerated view of the coloration of silver nitrate. In solution it is a colorless liquid. When applied no coloration is observed, but, after a few moments it discolors. Nitric acid is set free and combines, to a limited extent, with the lime salts of the tooth. The silver is precipitated on the surface, and not in the tubules, as an oxid, which becomes inert as soon as its action is limited. The idea of possible discoloration should not stand in the way of its use at all.

Dr. A. W. Harlan, Chicago, said that he made many experiments with teeth set in wax and plaster, and in the jaw itself, to test the penetrability of coagulating agents. A solution of silver nitrate will not penetrate the tubules to any appreciable extent; certainly not enough to cause discoloration of the tooth. The specimens passed around show that the oxide does not reach the cementum. He said he had a number of teeth imbedded, in which he had sealed nitrate of silver solution in 1894, but which he had not yet opened. He had any number of teeth in which the essential oils had been sealed. His experiments in this line have been very extended, and he knew what he was talking about. Chloride of zinc as soon as satisfied with water, stops its action. You can not drive nitrate of silver through the apex of a normal root, for when it comes in contact with the tissues at the end of the root it forms a coagulate and limits its own action. You will not get a permanent discoloration of the dentine with silver nitrate solution, for, on account of its coagulating properties, its action is limited. He was glad that Dr. Bethel and other Ohio men were experimenting in this direction.

Dr. H. L. Ambler, Cleveland, said nitrate of silver had been often used for superficial decay and to prevent further erosion by applying it to the affected surface of the tooth. He has found that when applied to an eroded surface by means of a minute piece of cotton saturated with the solution and the cataphoric current used, it penetrates deeper into the dentine and the effects are more lasting. Silver nitrate is superior to other agents, for it makes an insoluble compound with the albumen of the tissues. In root-canals just so far as the dentine is moistened with the nitrate you get the discoloration. He has experimented on pulps of freshly-extracted teeth and by means of the nitrate used cataphoretically they are thoroughly destroyed. It might prove an efficient means of devitalizing pulps.

